# Case Report: Targeting 2 Antigens as a Promising Strategy in Mixed Phenotype Acute Leukemia: Combination of Blinatumomab With Gemtuzumab Ozogamicin in an Infant With a *KMT2A*-Rearranged Leukemia

**DOI:** 10.3389/fonc.2021.637951

**Published:** 2021-02-26

**Authors:** Benoît Brethon, Elodie Lainey, Aurélie Caye-Eude, Audrey Grain, Odile Fenneteau, Karima Yakouben, Julie Roupret-Serzec, Lou Le Mouel, Hélène Cavé, André Baruchel

**Affiliations:** ^1^ Department of Pediatric Hematology, University Robert Debre Hospital, Assistance Publique Hôpitaux de Paris (APHP), Paris, France; ^2^ Department of Biological Hematology, University Robert Debre Hospital, APHP, Paris, France; ^3^ INSERM UMR_S1131, Institut de Recherche Saint-Louis, University of Paris, Paris, France; ^4^ Department of Genetics, University Robert Debre Hospital, APHP, Paris, France; ^5^ Transversal Unit for Therapeutic Patient Education, University Robert Debre Hospital, APHP, Paris, France; ^6^ University Institute of Hematology, University Saint-Louis Hospital, APHP, Paris, France

**Keywords:** mixed phenotype acute leukemia, infant, children, blinatumomab, gemtuzumab ozogamicin

## Abstract

Mixed phenotype acute leukemia (MPAL) accounts for 2-5% of leukemia in children. MPAL are at higher risk of induction failure. Lineage switch (B to M or vice versa) or persistence of only the lymphoid or myeloid clone is frequently observed in biphenotypic/bilineal cases, highlighting their lineage plasticity. The prognosis of MPAL remains bleak, with an event-free survival (EFS) of less than 50% in children. A lymphoid-type therapeutic approach appears to be more effective but failures to achieve complete remission (CR) remain significant. KMT2A fusions account for 75-80% of leukemia in infants under one year of age and remains a major pejorative prognostic factor in the Interfant-06 protocol with a 6 years EFS of only 36%. The search for other therapeutic approaches, in particular immunotherapies that are able to eradicate all MPAL clones, is a major issue. We describe here the feasibility and tolerance of the combination of two targeted immunotherapies, blinatumomab and Gemtuzumab Ozogamicin, in a 4-year-old infant with a primary refractory KTM2A-rearranged MPAL. Our main concern was to determine how to associate these two immunotherapies and we describe how we decided to do it with the parents’ agreement. The good MRD response on the two clones made it possible to continue the curative intent with a hematopoietic stem cell transplant at 9 months of age. Despite a relapse at M11 post-transplant because of the recurrence of a pro-B clone retaining the initial lymphoid phenotype, the child is now 36 months old, in persistent negative MRD CR2 for 12 months after a salvage chemotherapy and an autologous CAR T cells infusion, with no known sequelae to date. This case study can thus lead to the idea of a sequential combination of two immunotherapies targeting two distinct leukemic subclones (or even a single biphenotypic clone), as a potential one to be tested prospectively in children MPAL and even possibly all KMT2A-rearranged infant ALL.

## Introduction

Mixed phenotype acute leukemia (MPAL) accounts for 2%–5% of leukemia in children (1). It is a heterogeneous group of diseases whose immunophenotypic definition has changed over time (2–4). The current WHO classification recognizes two subclassifications of MPAL (biphenotypic and bilineal) with mostly similar clinical and genetics characteristics. MPAL are at higher risk of induction failure. Lineage switch (B to M or vice versa) or persistence of only the lymphoid or myeloid clone is frequently observed in biphenotypic/bilineal cases, highlighting their lineage plasticity. Prognosis of MPAL remains bleak, with an event-free survival (EFS) of less than 50% in children (1, 5–7). A lymphoid-type therapeutic approach appears to be more effective (6, 7) but failures to achieve complete remission (CR) remain significant. *KMT2A* fusions account for 75%–80% of leukemia in infants less than one year of age with typically an early pro-B CD10neg phenotype. Many cases express myeloid antigens (e.g., CD15/65, CD33, or weak MPO) but should not be considered MPAL unless unequivocal evidence of monoblastic differentiation (≥20% of blasts) or bright MPO expression, <5% of all cases; *KTM2A* rearrangement remains a major pejorative prognostic factor in the international Interfant-06 protocol and confers poor prognosis. The 6 years EFS for the low risk group (*KMT2A* germline patients) was 73.9% versus 36% for the *KMT2A*-rearranged group (8). The search for other therapeutic approaches, in particular immunotherapies that are able to eradicate all MPAL clones, is a major issue (9). We describe here the feasibility of the combination of two targeted immunotherapies in an infant with a primary refractory *KTM2A*-rearranged MPAL.

## Case Report

**Figure 1:**
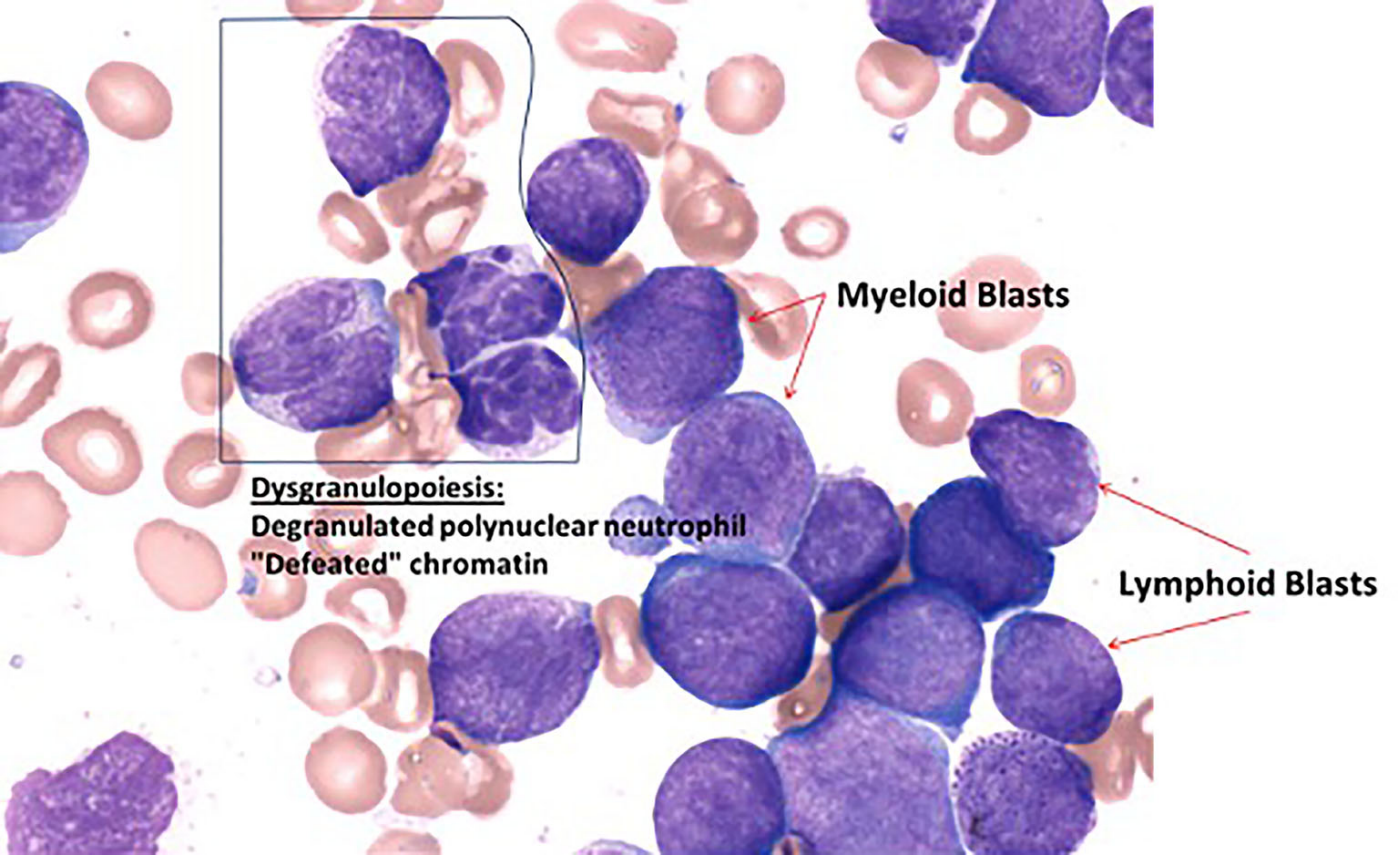
Cytological features of the bone marrow at diagnosis (May-Grünwald stain).

**Figure 2:**
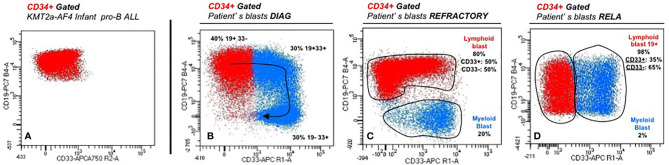
Immunophenotyping features at diagnosis **(A)**, following courses **(B, C)**, and relapse **(D)**.

**Figure 3:**
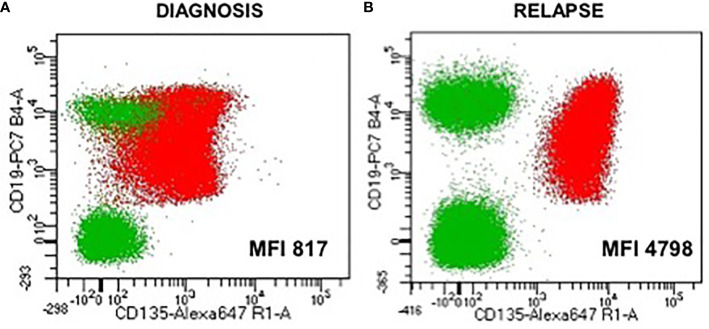
Expression of the FLT3 receptor (CD135) on the blast population at diagnosis and at relapse showing a six times more expression at relapse **(B)** than at diagnosis **(A)** *(*MFI, mean fluorescence intensity*)*.

Four-month-old little girl presented in February 2018 with asthenia, pallor, cutaneous hemorrhages, hepato-splenomegaly, and superior airways obstructive respiratory signs. Maximum white blood cells count was 762 G/L. Workup showed a spontaneous tumor lysis syndrome without renal failure with a grade 3 biological disseminated intravascular coagulation. HCoV-NL63 (non-SARS) coronavirus was found in the nasopharyngeal swab. Bone marrow was rich, infiltrated by 80% of lymphoblasts and 9% of resembling myeloblasts with fine granulations but peroxidase-negative. Atypical dysgranulopoiesis was also noted (**Figure 1**). Flow cytometry (FCM) showed two distinct CD34+ CD135+ (*FLT3*-R) populations evoking a MPAL (B/myeloid) diagnosis according to the WHO 2016. Pro-B clone (70% of blasts) was CD19bright, CD22dim partial, cCD79a partial, CD20-, CD24dim without surface Kappa/Lambda or cytoplasmic mu-chain and co-expressed on some blasts (30% of CD19+) several myeloid antigens (CD11c/CD64/CD15/CD33). Monocytic clone (30%) was CD19-, cCD79a-, MPO-, CD33/11b/65/15/11c/64bright with partial CD14 (15%) and partial co-expression of lymphoid markers CD22 and CD24 showing a continuum of expression/differentiation between the “pure” lymphoid and myeloid clones (**Figure 2A**). FISH evidenced *KTM2A* rearrangement in 93% of the nuclei. The presence of a *KMT2A-AFF1* (also known as *MLL-AF4*) fusion was confirmed using RT-PCR. A missense mutation of the *FLT3* gene (43% allelic frequency) was found (**Figure 3A**). No deletion or duplication was found using MLPA (MRC HOLLAND kit P335-C1). Cerebrospinal fluid status was CNS3.

The child was treated according to the recommendations of the high-risk arm of the Interfant-06 protocol. A poor prednisone response was observed at D8 (blasts 127.9 G/L). The CSF was normalized after three intrathecal injections. Unfortunately, CR was not achieved at the end of induction with a M2 bone marrow (9% blasts), progressing to a M3 bone marrow (32% blasts) 15 days after beginning the IB consolidation phase (cyclophosphamide/cytarabine/6-mercaptopurine). A course of high-dose cytarabine-based chemotherapy (2.25 g/m^2^ bid for 3 days) with amsidine (75 mg/m^2^ per day for 3 days) failed with FCM showing the persistence of both B-lymphoid (80%) and myeloid (20%) blasts (**Figure 2B**).

After having obtained the informed consent of the parents and the authorization of the French national drug safety agency (ANSM), multidisciplinary team decision was to attack specifically each of the two phenotypically distinct leukemic clones with a combination of Blinatumomab (BLIN) and Gemtuzumab Ozogamicin (GO). BLIN was introduced first in May 2018 at a dose of 5 microg/m^2^/day for 7 days and then increased at 15 microg/m^2^/day. GO (3 mg/m^2^) was added at D11, D15, D18 with triple intrathecal injections every 15 days (methotrexate, cytarabine, methylprednisolone). At D25, generalized seizures appeared in a febrile context, lasting for 5 min to stop BLIN. All radiological, biological, and microbiological documentations were negative. BLIN was resumed at D30 and continued until D73. One additional dose of GO 3 mg/m^2^ was administered at D44 for consolidation. The only other grade 3/4 toxicity was an undocumented febrile neutropenia; no liver toxicity including sinusoidal obstructive syndrome (SOS) was seen. In terms of response, CR1 was obtained at D38 BLIN/GO. Minimal residual disease (MRD) as undetectable at the threshold of 10^−4^ by FCM and 10^−5^ using genomic PCR established on the *KTM2A* breakpoint. This was confirmed at the time of discontinuation of BLIN (administered for 68 days in total). At that time, there were still circulating T-lymphocytes (CD3+ 780/mm^3^, CD4+ at 603/mm^3^) but no B-lymphocytes were seen as expected. This good response made it possible to continue the curative intent with a hematopoietic stem cell transplant in August 2018, i.e., at 9 months of age, after a conditioning regimen combining busulfan, fludarabine, and thiotepa. SOS prophylaxis consisted in defibrotide. A full donor chimerism was seen at mo1 and mo2. There was no acute GVHD or hepatic SOS. Ciclosporin A was stopped at mo3. MRD was negative again at M6 with both technics.

Unfortunately, a combined relapse occurred 11 months after HCST (child aged 21 months), with invasion of sinuses and orbits, a CNS2 CSF with 54% of lymphoid blasts in the bone marrow. FCM confirmed the recurrence of a pro-B clone retaining initial phenotype (CD19bright, CD10-, partial CD33 but no monocytic antigens), with *FLT3*-R overexpression as in diagnosis (**Figure 2C**). *KMT2A* rearrangement and the initial *FLT3* mutation were still detected with acquisition of a mono-allelic complete deletion of *IKZF1* (**Figure 3B**). Salvage treatment consisted of intensive chemotherapy combined with midostaurine BID and triple intrathecal infusions. Bone marrow and extra-medullary CR2 was obtained with undetectable *KTM2A* breakpoint MRD at level 10^−5^. Autologous anti-CD19 CAR-T cells were administered 3 months after the relapse followed by a grade 2 cytokine release syndrome with hematological and neurological toxicities. The child is now 36 months old, in persistent negative MRD CR2 (at mo12 post-CAR T cells infusion), with no known sequelae to date.

## Discussion

Pediatric MPALs are rare (<5% of pediatric AL cases), with up to 15% to 20% failures to achieve CR1. Very few data are available for children with *KMT2A*-rearranged MPAL (**Table 1**). Among 28 children treated for a MPAL with lymphoid-type treatment between 1996 and 2006 in the Czech Republic, 3 had a *KMT2A/AFF1* rearrangement, only one being alive after HSCT in CR2 (1). In a more recent series of 39 pediatric MPAL cases treated in Poland from 2007 to 2018, four cases presented with a *KMT2A* rearrangement but no details are provided regarding their specific treatment and outcome (7). An international cooperative study (18 centers participating in the iBFM-AMBI2012 study) looked at the therapeutic strategies and the outcome of 233 children with ambiguous lineage acute leukemia (ALAL) between 2002 and 2015 (176 single-population ALAL, 45 bilineal ALAL, 12 undifferentiated). Fifteen of 233 patients had a *KMT2A/AFF1* rearrangement and 11 had another type of *KMT2A* rearrangement. These cases most often aggregate in the group of patients who received a lymphoid and myeloid oriented “hybrid” treatment with a 5y EFS of only 28% (6).

**Table 1:** *KMT2A*-rerranged MPAL cases from literature and their treatment approach.

References	N of pts	N of KMT2A-r pts	Type of therapy	Survival
Mejstrikova et al. (1) Haematologica	28	**3** (*KMT2A/AFF1 : 3*)	Lymphoid type (interfant 2003; POG9407)	1/3
Hrusak et al. (6) Blood	233	**26** (*KMT2A/AFF1: 15 KMT2A*-r other: 11)	Hybrid myeloid/lymphoid type	5 y EFS **28%^+/−14%^**
Zając-Spychała et al. (7) Haematologica	39	**4**	NA	NA

New concepts and/or therapies and are thus needed for MPAL. BLIN is a commercialized bispecific CD19/CD3 monoclonal antibody. It has been tested for safety and efficacy in children with relapsed/refractory ALL with initial response rates of around 39%. Among 70 patients who received the recommended dosages (15 µg/m^2^/day after a week at 5 µg/m^2^/day), the CR rate was 56% among patients with <50% bone marrow blasts at baseline versus 33% among those with 50% or more blasts (10). In term or MRD response, among the 27 children who obtained a CR within the first two cycles, 14 (52%) achieved a complete MRD response, 13 (48%) by day 15 of cycle 1 (10). This was confirmed in a study comparing BLIN versus historical standard therapy in pediatric R/R Ph-negative B-cell precursor ALL (11). In infants less than 2 years of age, the response rate is 60% (6/10) with similar tolerance (10). GO is a commercialized humanized anti-CD33 monoclonal antibody coupled to the cytotoxic antibiotic calicheamicin causing DNA damage after endocytosis. This antibody has been shown to be effective in pediatric AML, including infants with similar and especially reasonable hepatic side effects (12, 13). Our main concern was to determine how to associate these two immunotherapies, one targeting the B lymphoid component, the other one targeting the myeloid component of the MPAL. BLIN allows the cytotoxic T lymphocytes to be engaged with malignant and benignant B cells, resulting in B cell lysis and T cell expansion. It needs preserved functional effector T-cells. GO, despite being a targeted chemotherapy, could precisely destroy those effectors. This is why we decided to first introduce BLIN alone, to induce the T–cell expansion and action on the B lineage component of the clone. Then GO was combined from D11 of the onset of BLIN, in a fractionated regimen we previously described as tolerated and effective in children with AML, at a dose of 3 mg/m^2^ three times in a week as monotherapy and in combination with cytarabine (14).

The history of our patient highlights the interest of a combined immunotherapy approach tailored to target immunophenotypic markers in MPAL. Indeed, lymphoid and myeloid clones, resistant to conventional chemotherapy, could be controlled by combining two drugs with a curative intent in this patient with a refractory disease. This case report can thus lead to the idea of a sequential combination of two immunotherapies targeting two distinct leukemic subclones (or even a single biphenotypic clone), as a potential one to be tested prospectively in children MPAL and even possibly all *KMT2A*-rearranged infant ALL.

## Data Availability Statement

The original contributions presented in the study are included in the article/supplementary material. Further inquiries can be directed to the corresponding author.

## Author Contributions

BB reviewed the literature, collected, and analyzed data and wrote the manuscript. BB and AB designed the case report. EL, AC-E, and OF provided biological data. All authors contributed to the article and approved the submitted version.

## Conflict of Interest

The authors declare that the research was conducted in the absence of any commercial or financial relationships that could be construed as a potential conflict of interest.
